# Endometrial Carcinoma Diagnosis: Use of FIGO Grading and Genomic Subcategories in Clinical Practice: Recommendations of the International Society of Gynecological Pathologists

**DOI:** 10.1097/PGP.0000000000000518

**Published:** 2018-12-14

**Authors:** Robert A. Soslow, Carmen Tornos, Kay J. Park, Anais Malpica, Xavier Matias-Guiu, Esther Oliva, Vinita Parkash, Joseph Carlson, W. Glenn McCluggage, C. Blake Gilks

**Affiliations:** Department of Pathology, Memorial Sloan Kettering Cancer Center, New York (R.A.S, K.J.P.); Department of Pathology, Stony Brook Hospital, SUNY, Stony Brook (C.T.), New York; Department of Pathology, The University of Texas MD Anderson Cancer Center, Houston, Texas (A.M.); Pathology Department, University Hospital Arnau de Vilanova and University Hospital of Bellvitge, Biomedical Research Institute, and Bellvitge Biomedical Institute, University of Lleida, CIBERONC, Spain (X.M.-G.); Department of Pathology, Massachusetts General Hospital, Boston, Massachusetts (E.O.); Department of Pathology, Yale School of Medicine and the Yale School of Public Health, New Haven, Connecticut (V.P.); Department of Oncology-Pathology, Karolinska Institutet and Department of Pathology and Cytology, Karolinska University Hospital, Stockholm, Sweden (J.C.); Department of Pathology, Belfast Health and Social Care Trust, Belfast, UK (W.G.M.); Department of Pathology and Laboratory Medicine, Vancouver General Hospital and University of British Columbia, Vancouver, BC, Canada (C.B.G.)

**Keywords:** Endometrial cancer, FIGO grade, The Cancer Genome Atlas, TCGA, Genomic subtype

## Abstract

In this review, we sought to address 2 important issues in the diagnosis of endometrial carcinoma: how to grade endometrial endometrioid carcinomas and how to incorporate the 4 genomic subcategories of endometrial carcinoma, as identified through The Cancer Genome Atlas, into clinical practice. The current International Federation of Gynecology and Obstetrics grading scheme provides prognostic information that can be used to guide the extent of surgery and use of adjuvant chemotherapy or radiation therapy. We recommend moving toward a binary scheme to grade endometrial endometrioid carcinomas by considering International Federation of Gynecology and Obstetrics defined grades 1 and 2 tumors as “low grade” and grade 3 tumors as “high grade.” The current evidence base does not support the use of a 3-tiered grading system, although this is considered standard by International Federation of Gynecology and Obstetrics, the American College of Obstetricians and Gynecologists, and the College of American Pathologists. As for the 4 genomic subtypes of endometrial carcinoma (copy number low/p53 wild-type, copy number high/p53 abnormal, polymerase E mutant, and mismatch repair deficient), which only recently have been identified, there is accumulating evidence showing these categories can be reproducibly diagnosed and accurately assessed based on biopsy/curettage specimens as well as hysterectomy specimens. Furthermore, this subclassification system can be adapted for current clinical practice and is of prognostic significance independent of conventional variables used for risk assessment in patients with endometrial carcinoma (eg, stage). It is too soon to recommend the routine use of genomic classification in this setting; however, with further evidence, this system may become the basis for the subclassification of all endometrial carcinomas, supplanting (partially or completely) histotype, and grade. These recommendations were developed from the International Society of Gynecological Pathologists Endometrial Carcinoma project.

This review focuses on the grading of endometrial endometrioid carcinoma (EEC) and the emerging molecular classification of endometrial carcinoma. In this review, we will discuss the pertinent literature and make recommendations as appropriate. At the end of each section, we will identify unresolved issues for which there are currently insufficient data to make recommendations, with the hope that these issues will be investigated in future research. These recommendations were developed by the authors as part of the International Society of Gynecologic Pathologists Endometrial Carcinoma Project.

## TOPIC 1: HOW TO APPLY THE INTERNATIONAL FEDERATION OF GYNECOLOGY AND OBSTETRICS (FIGO) GRADING SYSTEM FOR EEC

According to current practice standards, EECs are assigned a FIGO grade based on the degree of glandular differentiation. Grade 1 tumors exhibit ≤5% solid nonglandular, nonsquamous growth; grade 2 tumors from 6% to 50%; and grade 3 tumors >50% 1–3. The presence of marked cytologic atypia increases the grade 1 level. Since mucinous adenocarcinomas of the endometrium are closely related to endometrioid carcinomas, it is reasonable to use FIGO grade for those carcinomas as well. However, FIGO grading should NOT be used when endometrioid or mucinous differentiation is in doubt or cannot be established. All of the other endometrial tumor types carry an intrinsic tumor grade (i.e. serous, clear cell, and undifferentiated carcinomas and carcinosarcomas are high grade). Strategies for grading mixed epithelial tumors will be discussed later in this review and in another review in this issue.

**FIG. 1:**
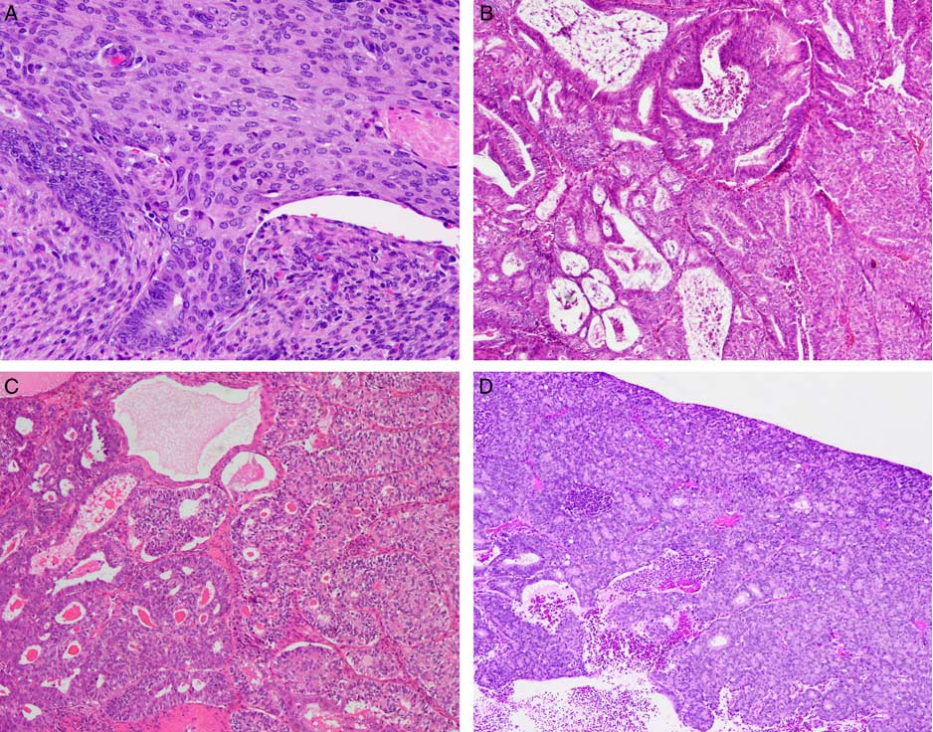
Examples of low-grade and high-grade endometrial endometrioid carcinomas (EEC): (A) low-grade EEC [International Federation of Gynecology and Obstetrics (FIGO) grade 1) with extensive squamous differentiation that does not qualify as “solid” for the purposes of grading; (B) low-grade EEC (FIGO grade 2) with <50% solid nonsquamous growth; (C) low-grade EEC (FIGO grade 2) with <50% solid nonsquamous growth; and (D) high-grade EEC with a microacinar growth pattern that qualifies as “solid growth.” The presence of microacini should not be considered “glandular” for the purposes of assigning binary or FIGO grade.

In clinical practice, determining FIGO grade is not always clear-cut. On the basis of personal experience and anecdotes accrued during participation in the Gynecologic Oncologic Group Pathology section, it appears that many pathologists consider tight, small microacini with barely visible lumens as solid growth, although FIGO grading rules do not specifically discuss this and some pathologists characterize such patterns as “glandular” (Fig. 1). For the purposes of grading, we endorse that a confluent microacinar pattern constitutes “solid” growth, although this is not evidence based. As stated in the FIGO criteria, squamous differentiation should be discounted as evidence of solid growth, but there are inevitable problems with grading tumors containing solid growth that resembles immature squamous epithelium and tumors that feature transitions between nonkeratinizing squamous epithelium and spindle cell change. It is reasonable to adjudicate these types of cases by paying attention to the nuclear grade, first in the glandular component and then, if that approach is not informative, in the solid component. We designate a tumor as FIGO grade 3 if the solid areas resemble poorly differentiated nonkeratinizing squamous cell carcinoma.

Another common problem in grading concerns the degree and extent of nuclear atypia that is sufficient to upgrade a tumor from 1 FIGO category to another 4,5. The philosophy underlying our approach to this problem is that discordance between architectural grade and nuclear grade should be uncommon in endometrioid adenocarcinomas. The first step is to ensure the nuclear features are sufficiently atypical. An easy guideline is to ask yourself, “Is this focus easily appreciated on scanning or intermediate power examination?” and “Is the atypia so bad that I would consider it grade 3 on a 3-point scale?” If the answers are “yes,” you should sample the tumor extensively to determine whether the finding is limited only to a few glands. If the change is diffuse, it is advisable to at least question whether part or all of the tumor could be a serous carcinoma or a clear cell carcinoma, instead of an endometrioid carcinoma 6. It is acceptable to move such a tumor from 1 FIGO grade to another only after determining that the tumor is indeed endometrioid throughout. A mixed epithelial carcinoma with an endometrioid component should be diagnosed when the cytologically atypical area is determined to be serous or clear cell on further study. One should not upgrade endometrioid carcinomas containing only a few glands with atypical nuclei, especially if it took a prolonged search at high-power magnification to recognize them. We endorse the severe nuclear atypia qualification described by Zaino et al. 4, that is, that severe nuclear atypia in the majority of cells (>50%) is required to upgrade a grade 1 or 2 EEC. “Upgrading” a case based on nuclear features is only rarely prudent, as in most cases, tumors are upgraded inappropriately (nuclear atypia is mild-moderate and diffuse, or severe and only focally found) or are not endometrioid at all.

Classification and regression tree statistical analyses have demonstrated that after tumor stage, the next most informative prognostic division in EEC is between high-grade (grade 3) and low-grade (grade 1/2) tumors 7,8. Although it is reported that there is a small but statistically significant difference in survival of up to 5% between clinically low-stage grades 1 and 2 EECs 9, this has not been consistently demonstrated. Therefore, the key consideration in tumor grading at hysterectomy should be to identify the presence of grade 3 EEC or any other high-grade components that may adversely affect patient prognosis, and not in trying to distinguish between grades 1 and 2 EEC. We have designated this system as “binary FIGO,” which entails combining grades 1 and 2 tumors into a low-grade category and grade 3 tumors into a high-grade category 10,11. Said another way, EECs with low nuclear grade and harboring ≤50% solid tumor components would be considered “low-grade EEC” (Fig. 1). This is in keeping with current risk assessment/treatment guidelines, such as those of the National Comprehensive Cancer Network (NCCN), European Society of Medical Oncology 12, the Mayo Clinic 13, and Memorial Sloan Kettering Cancer Center (MSK) in which grades 1 and 2 EECs are managed the same way. Although NCCN (NCCN guideline version 1.2018) still recognizes 3 EEC grades, the staging and therapeutic guidelines for grades 1 and 2 are very similar. Differences in the risk of lymph node metastasis in grades 1 and 2 EEC are most pronounced when decisions for staging are undertaken by evaluating biopsy or curettage material only. As will be discussed subsequently, the distinction between grades 1 and 2 is unimportant in the setting of planned lymphadenectomy or sentinel lymph node mapping, and distinguishing between these grades does not stratify patients who have had comprehensive surgical staging into different risk categories.

A literature review of various proposed binary grading schemes (summarized in Table 1 and a recent review on grading EEC) 1 reveals that EECs can readily be divided into either a low-risk or high-risk prognostic group based on a number of factors associated with increased tumor aggressiveness; the different grading systems show only slight disagreement over the distribution of a small number of gray-area cases between the 2 main prognostic groups. All of the proposed 2-tiered grading systems have been shown to be equal or superior to the current 3-tiered FIGO system in terms of interobserver variability kappa score, and the binary FIGO system, in the studies in which it was assessed, consistently exceeded the 3-tiered FIGO system. The binary FIGO system also performs as well as or better in terms of prognostication, compared with other binary grading schemes, and has the added advantage of being based on the currently used FIGO grading system (i.e. practicing pathologists are already familiar with the system’s criteria). An assessment of the potential clinical impact of the adoption of a binary EEC grading system is complicated by variations in clinical patient management, as well as the absence of a widely accepted consensus on the indications for extensive surgical staging in patients with a preoperative diagnosis of low-grade EEC. Soslow and colleagues recently analyzed a departmental database of clinical stage 1 EECs (n=1544) to better understand relationships between preoperative tumor grade, depth of myometrial invasion, and the risk of positive sentinel lymph nodes. The analysis demonstrated that while grade 1 tumors were associated with a significantly lower rate of lymph node metastases compared with grade 2 tumors overall (6.6% vs. 11.6%; *P*=0.003), the difference disappeared after adjusting for depth of myometrial invasion (Table 2) 1. Those findings further endorse the view that preoperative tumor grade alone should not be used to assess the indication for extensive surgical staging. The use of preoperative tumor grade alone to select patients for lymphadenectomy is clearly not supported by the evidence and does not reflect the complex interplay between the various pathologic parameters. In summary, we propose that it would be appropriate to adopt a binary FIGO system for grading EEC by combining the grades 1 and 2 categories into a single low-grade category in biopsy or curettages when comprehensive surgical staging is planned and in hysterectomy specimens. For patients desiring a fertility-sparing therapeutic approach, it will continue to be necessary to distinguish grades 1 and 2 based on currently used clinical criteria for conservative, hormonal therapy whereby grade 1 tumors can be considered for this approach and grade 2 tumors will generally not (NCCN guideline version 1.2018). We also propose to retain the severe nuclear atypia qualification, based on the work of Zaino et al. 4, whereby severe nuclear atypia in the majority of cells (>50%) in an architecturally low-grade EEC would lead to a high-grade designation. This proposed system should be beneficial in terms of simplification and diagnostic reproducibility.

**TABLE 1:**
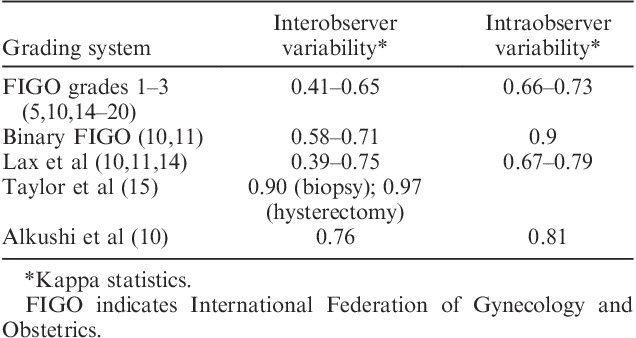
Interobserver and intraobserver variability of different grading systems

**TABLE 2:**
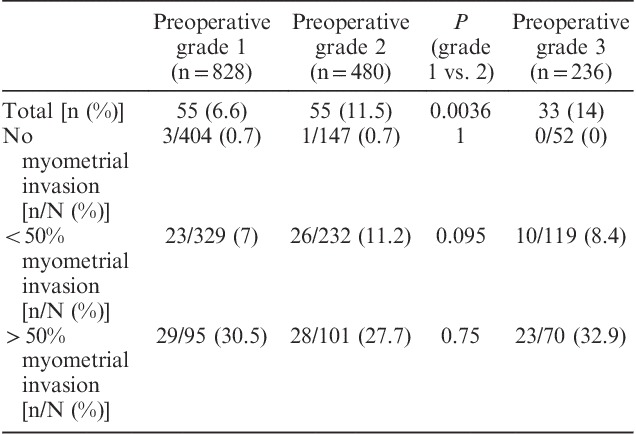
Rate of lymph node metastases in clinical stage 1 endometrial carcinoma in terms of preoperative tumor grade and depth of myometrial invasion (DMI) on hysterectomy: apparent differences in nodal positivity rates between grades is lost when adjusted for DMI

## RECOMMENDATIONS

- Discuss individual clinician’s preferences for moving to a binary grading scheme, while emphasizing its potential benefits.
- As a bridge toward uniform adoption of the binary FIGO grading scheme, it is acceptable to report both the binary grade and the FIGO grade [i.e. low-grade EEC (FIGO grade 2)].
- Going forward, we recommend the adoption of the binary FIGO grading system, when appropriate. Existing reporting data sets/synoptic templates/checklists for reporting endometrial carcinoma will need to be revised and updated.

## UNRESOLVED ISSUES

Anticipating the future incorporation of The Cancer Genome Atlas (TCGA) classification system into endometrial cancer diagnosis and prognostication, it remains uncertain whether grading will remain an important clinical discriminator for tumors, especially in the polymerase E (POLE) category.

## TOPIC 2: HOW TO SYNTHESIZE GENOMICALLY DEFINED TYPES OF ENDOMETRIAL CARCINOMA WITH THEIR MORPHOLOGIC, IMMUNOPHENOTYPIC, AND CLINICAL CHARACTERISTICS

For the purposes of this review, FIGO grade 3 endometrioid, serous, and clear cell carcinomas will be considered “high-grade endometrial carcinomas” (HGECs). Undifferentiated and dedifferentiated carcinomas and carcinosarcomas are dealt with elsewhere in this review series.

Various studies have shown that an HGEC diagnosis is not highly reproducible 21,22. This implies that: (1) diagnostic criteria are insufficiently detailed for practical use; (2) and/or diagnostic criteria were developed empirically and may not be evidence based; (3) and/or uniform diagnostic criteria are not utilized in practice. This lack of reproducibility is significant in that the reported clinicopathologic and molecular data concerning HGEC may not be readily interpreted because of differences in case series based on inconsistent diagnoses. Examples of the latter problem are the lack of consensus regarding whether grade 3 endometrioid carcinomas are “type II endometrial carcinomas” and whether they are as aggressive as serous carcinomas.

**FIG. 2:**
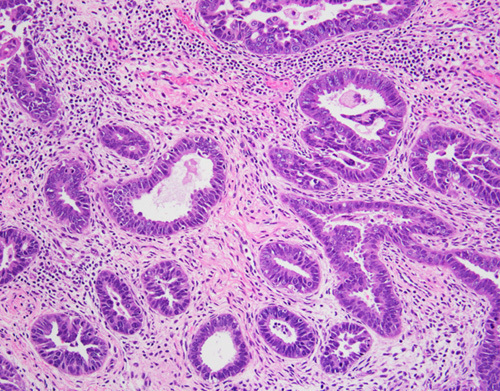
Serous carcinoma with a glandular pattern. Serous differentiation can be recognized at intermediate-power magnification by the presence of cells with high nuclear-to-cytoplasmic ratios and at high power by the presence of enlarged and atypical nuclei. Confirmatory endometrioid features are lacking. The diagnosis can be confirmed with a p53 immunohistochemical stain, which shows mutation-type staining in at least 90% of serous carcinomas. Although mutation-type p53 staining can be present in endometrioid carcinomas, almost all are International Federation of Gynecology and Obstetrics grade 3 carcinomas, which glandular serous carcinomas do not resemble.

There have been a number of attempts to refine diagnostic criteria using evidence-based approaches. Among other groups, the MSK group used immunohistochemical and clinical evidence to describe gland-forming serous carcinomas that are distinct from FIGO grades 1 and 2 endometrioid carcinomas 4,23 (Fig. 2); the MD Anderson group used supporting clinical evidence to separate undifferentiated carcinomas from grade 3 endometrioid carcinomas; 24,25 the Massachusetts General Hospital group used supporting clinical evidence to separate corded and hyalinized endometrioid carcinomas from carcinosarcomas; 26 and the Vancouver group used a genomically based classifier to refine diagnostic criteria that more precisely separate grade 3 endometrioid carcinoma with a papillary/villoglandular or solid architectural pattern from serous carcinomas with similar architecture 27. These latter criteria also have clinical relevance.

Publication of the TCGA endometrial carcinoma data set has provided pathologists a unique opportunity to further refine diagnostic criteria for serous and grade 3 endometrioid carcinomas 28. These data do not include information about clear cell carcinoma, undifferentiated/dedifferentiated carcinoma, or carcinosarcoma.

**FIG. 3:**
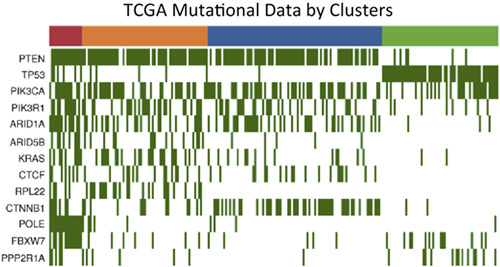
The Cancer Genome Atlas (TCGA) mutational data arranged by genomic clusters. Data from TCGA showing that *TP53* mutation analysis is a sensitive and specific method for distinguishing between copy number high and copy number low tumors. Note, however, that *TP53* mutations are also seen in polymerase E (POLE) and microsatellite instability-high (MSI-H) tumors.

Unlike the Bokhman “type I versus type II” classification of endometrial carcinoma, TCGA data indicated 4 genomic types of endometrial carcinoma. These subcategories of endometrial carcinoma were identified based on their genomic architecture. They include the following: *POLE* mutated/ultramutated; microsatellite instability-high (MSI-H)/hypermutated; copy number low (conceptually similar to “type I” endometrial carcinoma); and copy number high (presence of many genomic amplifications and/or deletions), also known as “the serous-like group,” which is similar to “type II” endometrial carcinoma. The copy number low group has the lowest mutation burden, whereas the hypermutated and ultramutated groups have, on average, 10-fold and 100-fold more mutations, respectively; the higher mutation burdens in these latter 2 groups are secondary to defects in mismatch repair (MMR) and POLE-mediated DNA repair, respectively. Note that mutations in *TP53* were a distinguishing feature, when comparing copy number high versus copy number low groups (Fig. 3), once POLE and MSI tumors were excluded.

The overwhelming majority of low-grade (FIGO grades 1 and 2) endometrioid carcinomas map to the copy number low and MSI-H categories. Grade 3 endometrioid carcinomas are highly heterogenous, as they are found in every genomic category, and are least represented in the copy number low group. Endometrioid carcinomas mostly have an endometrioid genomic profile, with or without *TP53* mutation/high copy number alterations, whereas serous carcinomas have *TP53* mutations/high copy number alterations without additional mutations characteristic of endometrioid carcinomas.

Assigning a tumor to the serous category requires the presence of a *TP53* mutation/abnormal “mutation pattern” p53 immunostaining, the absence of endometrioid-type mutations (although this point is debatable, as some low-grade endometrioid carcinomas with *PTEN* mutations acquire *TP53* mutations during tumor progression, along with “serous” morphology), and the presence of high copy number alterations. All serous carcinomas map to the copy number high, “serous-like” category, as do approximately one quarter of grade 3 endometrioid carcinomas. Although TCGA data are incomplete, they were sufficient to generate a Kaplan-Meier survival curve showing that POLE carcinomas have outstanding clinical outcomes irrespective of grade and perhaps stage. MSI-H and copy number low tumors have intermediate clinical risk, and the serous-like group, unsurprisingly, has the worst outcomes.

The first approach to extracting this information for practical diagnostic use was to describe the morphology of endometrioid carcinomas in each group. Although there are many characteristic features of tumors from the POLE 29,30 (peritumoral lymphocytes, tumor infiltrating lymphocytes, and intratumoral heterogeneity, tumor giant cells), MSI-H 31,32 (peritumoral lymphocytes, tumor infiltrating lymphocytes, and intratumoral heterogeneity), and serous-like groups (diffuse high nuclear grade), there is a general feeling that these features are incomplete, partly overlapping, and may not be able to be applied reproducibly. A particular problem that is not adequately addressed with a morphology-only approach is the existence of morphologically ambiguous tumors that reside in the POLE, MSI-H, and serous-like categories (but not the copy number low category) 33, and many POLE endometrioid carcinomas have components or subclones that are histologically similar to serous carcinomas. Given the particular heterogeneity of grade 3 endometrioid carcinomas, we were faced with the following questions and answers:

- Are serous-like grade 3 endometrioid carcinomas, as genetically defined, really endometrioid based on morphologic features/light microscopic examination?
  - In some cases, they can show unequivocally endometrioid morphologic features, for example, squamous differentiation 34. Many are histologically ambiguous, as mentioned above, making the distinction between serous-like grade 3 endometrioid and serous carcinomas difficult or impossible; this also raises the question of whether there is a justification for attempting to separate these tumors.

- Are serous-like grade 3 endometrioid carcinomas clinically interchangeable with serous carcinomas?
  - *TP53-*mutated grade 3 endometrioid carcinomas, compared with those lacking *TP53* mutation, recur more frequently (T. Bosse et al., *Am J Surg Pathol* 2018, in press) 35. Detailed analyses indicate that *TP53-*mutated grade 3 endometrioid carcinomas (i.e. serous*-*like grade 3 endometrioid carcinomas) have a high-risk profile, with only rare exceptions. Although there are few such cases, it appears that *TP53*-mutated grade 3 endometrioid carcinomas from the POLE category retain the clinically low-risk profile of POLE endometrioid carcinomas that lack a *TP53* mutation. There are sparse data suggesting that serous-like grade 3 endometrioid carcinomas metastasize to the peritoneum less frequently than serous carcinomas, but this requires validation. Currently, there is no compelling evidence to support that serous-like grade 3 endometrioid carcinomas are significantly clinically different than serous carcinomas, and unless there is improvement in diagnostic reproducibility for these 2 categories, it is difficult to envision such a distinction being a variable that can be used to guide patient treatment decisions.
- Can p53 immunohistochemistry alone be used to identify those grade 3 endometrioid carcinomas that belong to the copy number high group?
  - No. As aberrant p53 staining is not confined to tumors in the serous-like category, additional ancillary tests would be necessary to exclude a *TP53*-mutated POLE or MSI-H grade 3 endometrioid carcinoma. Of interest, many *TP53* mutations found in the POLE and MSI-H categories involve portions of the *TP53* gene that are not mutated in serous carcinomas 36, leading to imperfect correlation between *TP53* mutation and aberrant p53 staining in these groups of tumors.
- Is there a better assay than p53 immunohistochemistry to determine which grade 3 endometrioid carcinomas are serous-like?
  - p53 immunohistochemistry was compared with *TP53* mutational analysis and fluorescent in situ hybridization for copy number analysis and found to be equivalent in identifying the copy number high serous/serous-like group 36 (see below). Since aberrant p53 immunostaining is not synonymous with a copy number high tumor, other methods may prove to be superior surrogate markers of this genomic subtype. Aneuploidy, which is intuitively a good candidate marker of copy number high tumor, frequently was found to be present within the MSI-H and POLE categories, so testing to exclude MSI-H and POLE is, at this time, necessary before separation of copy number high and low tumors 37.

We used TCGA data to extract surrogate markers for the genome-wide analyses used to classify endometrial cancers into 1 of the 4 genomic categories, and then validated the classifier in an independent set of cases; this classifier, referred to as the ProMisE (Proactive Molecular Risk Classifier for Endometrial Cancer) classifier, includes MMR immunostaining (rather than MSI analysis, as was used in TCGA), sequencing of *POLE* exonuclease domains (rather than quantification of mutations genome wide) and p53 immunohistochemistry (rather than quantification of copy number alterations genome wide or *TP53* mutational analysis), and was able to recreate the 4 prognostically significant genomic subsets of endometrial carcinoma 36,38 (Table 3). An identical strategy independently used by the group from Leiden was applied to clinical trial cases and also demonstrated that the 4 molecular subtypes are of prognostic significance 39.

**TABLE 3:**
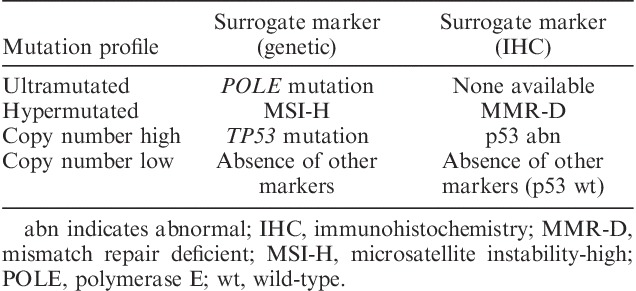
The 4 molecular subtypes of endometrial carcinoma

### Proposal

**FIG. 4:**
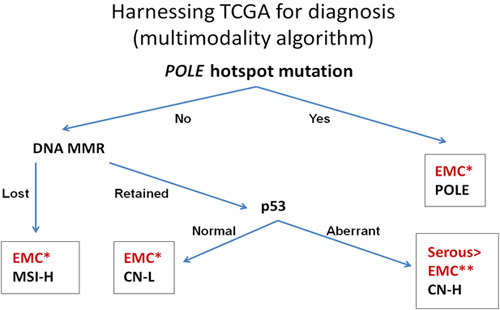
Harnessing The Cancer Genome Atlas (TCGA) data for clinical diagnosis. CN-H indicates copy number high (serous or serous-like endometrial carcinoma); CN-L, copy number low (type I EMC); EMC, endometrial carcinoma; MSI-H, microsatellite instability-high; POLE, polymerase E hotspot mutant tumor. EMC*: This may also be applicable to clear cell carcinomas. Most cases can be separated with review of hematoxylin and eosin slides. EMC**: This assay does not distinguish CN-H grade 3 endometrioid carcinoma or CN-H clear cell carcinoma from serous carcinoma.

On the basis of this information, we propose a multimodality classification system of HGECs that separates the 4 genomically defined groups of HGEC using only the following assays (Table 3 and Fig. 4): *POLE* mutational analysis and immunohistochemistry for p53 and the DNA MMR markers PMS2 and MSH6. As noted previously, this algorithm has been validated in 3 independent case series, including in Post Operative Radiation Therapy in Endometrial Carcinoma (PORTEC) clinical trial cases 36,38,39.

This multimodality molecular classification system may not be feasible in many pathology laboratories, especially in resource-restricted areas of the world. In resource-rich regions of the world, the main impediment to making this proposal the standard of care is not only buy-in from our clinical colleagues and the availability of *POLE* mutational testing, but also the recognition by regulatory authorities that this is a crucial, medically valid, and necessary assay (and in the United States, this means being worthy of reimbursement by insurance companies).

For laboratories that do not have the means to perform *POLE* mutational analysis, it will still be possible to identify the MMR-deficient (MMR-D) and p53 abnormal groups using immunostaining with a 3-marker panel (PMS2, MSH6, and p53). The ∼8% of endometrial cancers in the POLE group will not be identified, and separating these frequently histologically high-grade and FIGO stage I carcinomas from serous carcinomas will remain a problem. At this time, we do not know if the favorable prognosis of the histologically high-grade *POLE*-mutant endometrial cancers reflects indolent biology, effective most immunity, or a good response to treatment, and until this is resolved through clinical trials, conventional therapy, based on current risk assessment (eg, European Society of Medical Oncology guidelines), will continue to be implemented for these tumors, and their prognosis will remain excellent.

There is also some resistance to performing universal DNA MMR protein immunohistochemistry. The clinical argument for universal MMR testing is compelling, and this approach is recommended in the NCCN guidelines (NCCN guideline version 1.2018) for Lynch syndrome triage and in another review in this issue. There are other benefits to universal testing, as it may be used as an ancillary diagnostic test (i.e. to help exclude a serous carcinoma or to help assign endometrial rather than cervical primary site) and, in the setting of recurrent endometrial carcinoma, it provides therapeutic prediction for the use of immunotherapy.

What about clear cell carcinomas? Work from the MSK group indicates that endometrial clear cell carcinomas are just as heterogenous as grade 3 endometrioid carcinomas. There are *POLE* hotspot mutated, MSI-H, copy number low, and copy number high (serous-like) clear cell carcinomas, and those in the copy number low and serous-like clusters have significantly worse clinical outcomes compared with the others 40. Once this work is confirmed by other groups, we would provisionally propose that clear cell carcinomas be classified based on the same multimodality system as that of grade 3 endometrioid carcinomas.

### Related and Unresolved Issues

#### Terminology

Current treatment paradigms rely on the classic parameters of tumor histotype, especially the distinction between endometrioid versus nonendometrioid histology, and grade (i.e. FIGO grade as applied to endometrioid-type tumors). Therefore, any recommended terminology using the genomic classifier must continue to clearly include these parameters for the foreseeable future, although unpublished data from MSK and recently published data from Vancouver 36,38 indicate that prognosis is independent of histotype (in a multivariate model), and instead depends on stage, grade, and genomic classification. We would therefore recommend that reporting the genomic classifier be clearly noted, as well as the analyses used to assign the classification. For example:

Endometrioid endometrial carcinoma, FIGO grade 3. Genomic classifier group: Copy number high (p53 abnormal by immunostaining)

Or

Mixed endometrioid and serous carcinoma. Genomic classifier group: POLE (*POLE* exonuclease mutation identified by sequencing)

### When to Utilize the ProMisE Algorithm

Universal MMR testing should be performed on biopsy/curettage material in patients desiring fertility-sparing conservative medical management, since the recognition of Lynch syndrome in such cases would encourage prophylactic hysterectomy and bilateral salpingo-oophorectomy at the appropriate age. These tests should also be considered in biopsy/curettage material when treatment planning depends on genomic classification. This is not yet a standard practice, although selected gynecologic pathology teams are currently using this approach. It should also be noted that antigen preservation may be superior in biopsies/curettages compared with hysterectomy specimens, and that molecular classification based on the biopsy does accurately reflect the findings in the subsequent hysterectomy specimen 41,42.

### Tumor Grading

Tumor grading in the context of molecular subtype is a complex issue. FIGO grading is presumably only appropriate for tumors that are within the MSI-H/MMR-D or copy number low (also referred to as tumors with no specific molecular profile, as they lack *POLE* mutation, MMR deficiency, or abnormal p53 immunostaining) groups, as these most clearly correspond to the “classic” endometrioid tumors. FIGO grading in these 2 groups may provide the prognostic information that one expects from older, histology-based clinical studies, and would allow them to be provisionally placed into low-risk and high-risk groups, although this remains to be studied. In contrast, tumors in the copy number high/p53 abnormal group (serous-like) clearly belong in the “high-risk” group, in which no histologic grading is appropriate. There is likely no value in assigning grade to tumors in the POLE group, for which studies indicate a survival rate approaching 100%. A significant proportion of these tumors morphologically would be considered high grade and could potentially be treated with unnecessary lymphadenectomy and chemotherapy. The intersection of genomic classification, surgical staging, and delivery of adjuvant therapy is not yet resolved.

### Intratumoral Heterogeneity

Some researchers strongly believe that endometrial carcinomas are more heterogenous than their ovarian counterparts (i.e. more likely to contain true mixed tumors). Van Esterik et al. 43 demonstrated that this morphologic heterogeneity does not extend to the level of genomic abnormalities, that is, genomic subtype is uniform throughout.

Many tumors assigned to the MMR-D/MSI-H and POLE categories demonstrate significant intratumoral morphologic heterogeneity, whereas tumors in these groups and the p53 abnormal/copy number high cluster also typically show morphologic ambiguity 44,45 that gives rise to diagnostic problems 33. Hence, where does intratumoral heterogeneity stop and mixed epithelial carcinoma begin? Given emerging data 46 indicating that most mixed epithelial carcinomas represent different morphologic expressions of 1 clonal tumor, one is left with the impression that mixed epithelial carcinomas may not be diagnosed with any frequency in the future, apart from some exceptional situations, for example, grade 1 EEC giving rise to undifferentiated carcinoma. Also, what do we make of the assertion that any type II component of a mixed epithelial carcinoma places the tumor in a clinically high-risk category? It is probable that most “mixed epithelial carcinomas” with poor clinical outcomes represent heterogenous or morphologically ambiguous p53 abnormal/copy number high tumors. Other examples, however, likely represent heterogenous or ambiguous POLE or MSI-H tumors whose clinical behavior is baseline or favorable despite the histologically high-grade appearance of 1 portion of the tumor. These concepts need to be developed further and validated with good clinical, morphologic, and genomic data. For the time being, it is reasonable to continue to follow the ISGP recommendation that grading mixed epithelial carcinomas (such as mixed endometrioid and serous or clear cell carcinoma) be based on the highest grade component (discussed in another review in this issue). However, it also must be recognized this practice may not be clinically informative in MSI-H and POLE tumors.

## SUMMARY/RECOMMENDATIONS

- TCGA classification separates prognostically favorable from unfavorable types of FIGO grade 3 endometrioid carcinoma. Furthermore, it separates serous carcinoma from hitherto unknown types of clinically low-grade endometrial carcinomas that can morphologically mimic serous carcinoma to a substantial degree (eg, POLE, MMR-D). The classification is powerfully prognostically relevant and is independently associated with clinical outcomes, unlike histologic subtype assignment based on examination of hematoxylin and eosin slides alone.
- In laboratories undertaking genomic classification, this should be reported in conjunction with grade and histotype.

## UNRESOLVED ISSUES

- Since the practical application of TCGA classification relies on universal *POLE* gene sequencing and universal immunohistochemistry for p53 and 2 or 4 of the DNA MMR markers, reimbursement and financial issues need to be considered. We do not expect significant problems with implementation once regulatory authorities understand the potentially enormous clinical impact of these assays.
- Once regulatory and financial issues are resolved, the most efficient workflow for accomplishing the ancillary tests, interpreting them and incorporating them into a diagnostic report that synthesizes morphology, *POLE* genotype and immunophenotype will need to be defined.
- As accumulating data already suggest modifications to the “high-intermediate risk category” of uterus-confined endometrial carcinoma based on ProMisE/TCGA cluster assignment, clinical trials to validate its usefulness for clinical applications will be needed.
- It is uncertain whether it will continue to be necessary to distinguish serous-like (p53 abnormal/copy number high) FIGO grade 3 endometrioid, serous, and clear cell carcinomas.
- Although there are data indicating that the ProMisE classifier reliably assigns matched tumors from preoperative and hysterectomy specimens to the same genomic group 40,41, it is uncertain whether preoperative ProMisE/TCGA classification (using biopsy or curettage material) can be used to triage patients to different types of staging surgery. For example, patients with *POLE*-mutant tumors might not need comprehensive surgical staging. Similarly, patients with serous and serous-like carcinomas may not benefit from comprehensive surgical staging, since nearly all will require adjuvant chemotherapy, and it is possible that comprehensive surgical staging may only be applicable to the remaining categories.

## References

[1] Conlon N Leitao MM Jr Abu-Rustum NR Grading uterine endometrioid carcinoma: a proposal that binary is best. Am J Surg Pathol 2014;38:1583–7. 25229772 10.1097/PAS.0000000000000327

[2] Benda JA Zaino R Gynecologic Oncology Group Pathology Manual. Philadelphia, PA: Gynecologic Oncology Group; 1994 .

[3] Announcements. International Federation of Gynecology and Obstetrics (FIGO) stages—1988 revision. Gynecol Oncol 1989;35:125–7.

[4] Zaino RJ Kurman RJ Diana KL The utility of the revised International Federation of Gynecology and Oncology histologic grading of endometrial endometrioid carcinoma using a defined nuclear grading system. Cancer 1995;75:81–6. 7804981 10.1002/1097-0142(19950101)75:1<81::aid-cncr2820750114>3.0.co;2-f

[5] Nielsen AL Henrik KT Nyholm HCJ Evaluation of the reproducibility of the revised 1988 International Federation of Gynecology and Oncology (FIGO) grading system of endometrial cancers with special emphasis on nuclear grading. Cancer 1991;68:2303–9. 1913466 10.1002/1097-0142(19911115)68:10<2303::aid-cncr2820681033>3.0.co;2-y

[6] Garg K Soslow RA Strategies for distinguishing low-grade endometrioid and serous carcinomas of endometrium. Adv Anat Pathol 2012;19:1–10. 22156830 10.1097/PAP.0b013e318234ab36

[7] Barlin JN Zhou Q St. Clair CM Classification and regression tree (CART) analysis of endometrial carcinoma: seeing the forest for the trees. Gynecol Oncol 2013;130:452–6. 23774300 10.1016/j.ygyno.2013.06.009 PMC3748179

[8] Barlin JN Soslow RA Lutz M Redefining Stage I endometrial cancer incorporating histology, a binary grading system, myometrial invasion and lymph node assessment. Int J Gyn Cancer 2013;23:1620–8. 10.1097/IGC.0b013e3182a5055e PMC4405774 24126219

[9] Surveillance, Epidemiology, and End Results (SEER) Program stat fact sheets: endometrial cancer. Available at: http://seer.cancer.gov/statfacts/html/corp.html. Accessed July 3, 2017.

[10] Alkushi A Abdul-Rahman Z Lim P Description of a novel system for grading endometrial carcinoma and comparison with existing grading systems. Am J Surg Path 2005;29:295–304. 15725797 10.1097/01.pas.0000152129.81363.d2

[11] Scholten AN Smit VT Beerman H Prognostic significance and interobserver variability of histologic grading systems for endometrial carcinoma. Cancer 2004;100:764–72. 14770433 10.1002/cncr.20040

[12] Colombo N Preti E Landoni F Endometrial cancer: ESMO Clinical Practice Guidelines for diagnosis, treatment and follow-up. Ann Oncol 2013;24(suppl 6):vi33-8. 24078661 10.1093/annonc/mdt353

[13] AlHilli M Podratz K Dowdy S Risk-scoring system for the individualized prediction of lymphatic dissemination in patients with endometrioid endometrial cancer. Gynecol Oncol 2013;131:103–8. 23845691 10.1016/j.ygyno.2013.06.037

[14] Lax SF Kurman RJ Pizer ES A binary architectural grading system for uterine endometrial endometrioid carcinoma has superior reproducibility compared with FIGO grading and identifies subsets of advance-stage tumors with favorable and unfavorable prognosis. Am J Surg Path 2000;24:1201–8. 10976693 10.1097/00000478-200009000-00002

[15] Taylor RR Zeller J Lieberman RW An analysis of two versus three grades for endometrial carcinoma. Gynecol Oncol 1999;74:3–6. 10385544 10.1006/gyno.1999.5422

[16] Gemer O Uriev L Voldarsky M The reproducibility of histological parameters employed in the novel binary grading systems of endometrial cancer. Eur J Surg Oncol 2009;35:247–51. 18775628 10.1016/j.ejso.2008.07.010

[17] Kapucuoglu N Bulbul D Tulunay G Reproducibility of grading systems for endometrial endometrioid carcinoma and their relation with pathologic prognostic parameters. Int J Gynecol Cancer 2008;18:790–6. 17892460 10.1111/j.1525-1438.2007.01067.x

[18] Nedergaard L Jacobsen M Andersen JE Interobserver agreement for tumour type, grade of differentiation and stage in endometrial carcinomas. APMIS 1995;103:511–8. 7576566 10.1111/j.1699-0463.1995.tb01399.x

[19] Stefansson IM Salvesen HB Immervoll H Prognostic impact of histological grade and vascular invasion compared with tumour cell proliferation in endometrial carcinoma of endometrioid type. Histopathology 2004;44:472–9. 15139995 10.1111/j.1365-2559.2004.01882.x

[20] Guan H Semaan A Bandyopadhyay S Prognosis and reproducibility of new and existing binary grading systems for endometrial carcinoma compared to FIGO grading in hysterectomy specimens. Int J Gynecol Cancer 2011;21:654–60. 21543931 10.1097/IGC.0b013e31821454f1

[21] Gilks CB Oliva E Soslow RA Poor interobserver reproducibility in the diagnosis of high-grade endometrial carcinoma. Am J Surg Pathol 2013;37:874–81. 23629444 10.1097/PAS.0b013e31827f576a

[22] Han G Sidhu D Duggan MA Reproducibility of histological cell type in high-grade endometrial carcinoma. Mod Pathol 2013;26:1594–604. 23807777 10.1038/modpathol.2013.102

[23] Darvishian F Hummer AJ Thaler HT Serous endometrial cancers that mimic endometrioid adenocarcinomas: a clinicopathologic and immunohistochemical study of a group of problematic cases. Am J Surg Pathol 2004;28:1568–78. 15577675 10.1097/00000478-200412000-00004

[24] Altrabulsi B Malpica A Deavers MT Undifferentiated carcinoma of the endometrium. Am J Surg Pathol 2005;29:1316–21. 16160474 10.1097/01.pas.0000171003.72352.9a

[25] Silva EG Deavers MT Bodurka DC Association of low-grade endometrioid carcinoma of the uterus and ovary with undifferentiated carcinoma: a new type of dedifferentiated carcinoma? Int J Gynecol Pathol 2006;25:52–8. 16306785 10.1097/01.pgp.0000183048.22588.18

[26] Murray SK Clement PB Young RH Endometrioid carcinomas of the uterine corpus with sex cord-like formations, hyalinization, and other unusual morphologic features: a report of 31 cases of a neoplasm that may be confused with carcinosarcoma and other uterine neoplasms. Am J Surg Pathol 2005;29:157–66. 15644772 10.1097/01.pas.0000149704.89463.05

[27] Hoang LN McConechy MK Köbel M Histotype-genotype correlation in 36 high-grade endometrial carcinomas. Am J Surg Pathol 2013;37:1421–32. 24076778 10.1097/PAS.0b013e31828c63ed

[28] Cancer Genome Atlas Research Network, Kandoth C Schultz N Cherniack AD Integrated genomic characterization of endometrial carcinoma. Nature 2013;497:67–73. 23636398 10.1038/nature12113 PMC3704730

[29] Hussein YR Weigelt B Levine DA Clinicopathological analysis of endometrial carcinomas harboring somatic POLE exonuclease domain mutations. Mod Pathol 2015;28:505–14. 25394778 10.1038/modpathol.2014.143

[30] Bakhsh S Kinloch M Hoang LN Histopathological features of endometrial carcinomas associated with POLE mutations: implications for decisions about adjuvant therapy. Histopathology 2016;68:916–24. 26416160 10.1111/his.12878 PMC5650229

[31] Shia J Black D Hummer AJ Routinely assessed morphological features correlate with microsatellite instability status in endometrial cancer. Hum Pathol 2008;39:116–25. 17949789 10.1016/j.humpath.2007.05.022

[32] Garg K Leitao MM Jr Kauff ND Selection of endometrial carcinomas for DNA mismatch repair protein immunohistochemistry using patient age and tumor morphology enhances detection of mismatch repair abnormalities. Am J Surg Pathol 2009;33:925–33. 19238076 10.1097/PAS.0b013e318197a046

[33] Hussein YR Broaddus R Weigelt B The genomic heterogeneity of FIGO grade 3 endometrioid carcinoma impacts diagnostic accuracy and reproducibility. Int J Gynecol Pathol 2016;35:16–24. 26166718 10.1097/PGP.0000000000000212 PMC4934379

[34] Schultheis AM Martelotto LG De Filippo MR TP53 mutational spectrum in endometrioid and serous endometrial cancers. Int J Gynecol Pathol 2016;35:289–300. 26556035 10.1097/PGP.0000000000000243 PMC5087968

[35] Bosse T Nout RA McAlpine JN Molecular classification of grade 3 endometrioid endometrial cancers identifies distinct prognostic subgroups. Am J Surg Pathol 2018;42:561–568. 29505428 10.1097/PAS.0000000000001020 PMC5893364

[36] Talhouk A McConechy MK Leung S A clinically applicable molecular-based classification for endometrial cancers. Br J Cancer 2015;113:299–310. 26172027 10.1038/bjc.2015.190 PMC4506381

[37] Proctor L Pradhan M Leung S Assessment of DNA Ploidy in the ProMisE molecular subgroups of endometrial cancer. Gynecol Oncol 2017;146:596–602. 28647100 10.1016/j.ygyno.2017.06.020

[38] Talhouk A McConechy MK Leung S Confirmation of ProMisE: a simple, genomics-based clinical classifier for endometrial cancer. Cancer 2017;123:802–13. 28061006 10.1002/cncr.30496

[39] Stelloo E Nout RA Osse EM Improved risk assessment by integrating molecular and clinicopathological factors in early-stage endometrial cancer-combined analysis of the PORTEC cohorts. Clin Cancer Res 2016;22:4215–24. 27006490 10.1158/1078-0432.CCR-15-2878

[40] DeLair DF Burke KA Selenica P The genetic landscape of endometrial clear cell carcinomas. J Pathol 2017;243:230–41. 28718916 10.1002/path.4947 PMC5708127

[41] Talhouk A Hoang LN McConechy MK Molecular classification of endometrial carcinoma on diagnostic specimens is highly concordant with final hysterectomy: earlier prognostic information to guide treatment. Gynecol Oncol 2016;143:46–53. 27421752 10.1016/j.ygyno.2016.07.090 PMC5521211

[42] Kommoss S McConechy MK Kommoss F Final validation of the ProMisE molecular classifier for endometrial carcinoma in a large population-based case series. Ann Oncol 2018 PMID:29432521. [Epub ahead of print]. 10.1093/annonc/mdy058 29432521

[43] van Esterik M Van Gool IC de Kroon CD Limited impact of intratumour heterogeneity on molecular risk assignment in endometrial cancer. Oncotarget 2017;8:25542–51. 28424422 10.18632/oncotarget.16067 PMC5421949

[44] Soslow RA Endometrial carcinomas with ambiguous features. Semin Diagn Pathol 2010;27:261–73. 21309260 10.1053/j.semdp.2010.09.003

[45] Soslow RA High-grade endometrial carcinomas—strategies for typing. Histopathology 2013;62:89–110. 23240672 10.1111/his.12029

[46] Köbel M Meng B Hoang LN Molecular analysis of mixed endometrial carcinomas shows clonality in most cases. Am J Surg Pathol 2016;40:166–80. 26492180 10.1097/PAS.0000000000000536 PMC5029122

